# Safety of esketamine nasal spray: Analysis of post-marketing reports submitted to the FDA adverse event reporting system in the first year on the market

**DOI:** 10.1192/j.eurpsy.2021.410

**Published:** 2021-08-13

**Authors:** C. Gastaldon, E. Raschi, J. Kane, C. Barbui

**Affiliations:** 1 University Of Verona, WHO Collaborating Centre for Research and Training in Mental Health and Service Evaluation, Verona, Italy; 2 Neuroscience, Psychological And Psychiatric Science, Science Of Bio Movement, University of Verona, Verona, Italy; 3 Department Of Medical And Surgical Sciences, University of Bologna, Bologna, Italy; 4 Department Of Psychiatry, The Zucker Hillside Hospital, Northwell Health, Glen Oaks, NY, United States of America

**Keywords:** treatment-resistant depression, pharmacovigilance, esketamine, Suicidal risk

## Abstract

**Introduction:**

The approval of the esketamine nasal spray for treatment-resistant depression in March 2019 by the Food and Drug Administration (FDA), and few months later by the European Medicine Agency, triggered a vivid debate and many concerns, mainly because of the lack of convincing evidence on its efficacy and safety, based on the development programs, approval trials and few post-marketing trials.

**Objectives:**

We aimed to detect and characterize safety signals for esketamine, by analyzing relevant adverse events (AEs) reports in the FDA Adverse Event Reporting System (FAERS) database (March 2019-March 2020).

**Methods:**

We performed disproportionality analysis through the case/non-case approach: reporting odds ratios (ROR) and information components (IC) with 95% confidence intervals (95%CI) were estimated for esketamine-related AEs with at least four counts. We compared serious and non-serious AEs using non-parametrical tests.

**Results:**

The FAERS database registered 962 reports of esketamine-related AEs in one year. Signals (i.e., statistically significant disproportionality) were detected for several AEs, such as dissociation, sedation, feeling drunk, suicidal ideation and completed suicide. Signals for suicidal ideation, but not suicide attempt and completed suicide, remained significant when comparing esketamine to venlafaxine. The comparison of patients with serious vs. non-serious esketamine AEs revealed that females, patients receiving antidepressant polypharmacy, co-medication with antipsychotics, mood stabilizers, benzodiazepines or somatic medications were more likely to suffer from serious AEs.

**Conclusions:**

This real-world pharmacovigilance analysis detected signals of serious unexpected esketamine-related AEs, thus reinforcing current worries regarding esketamine safety/acceptability. Further real-world studies are urgently needed to unravel the safety profile of esketamine.

**Disclosure:**

No significant relationships.

